# Through-needle all-optical ultrasound imaging *in vivo*: a preclinical swine study

**DOI:** 10.1038/lsa.2017.103

**Published:** 2017-12-01

**Authors:** Malcolm C Finlay, Charles A Mosse, Richard J Colchester, Sacha Noimark, Edward Z Zhang, Sebastien Ourselin, Paul C Beard, Richard J Schilling, Ivan P Parkin, Ioannis Papakonstantinou, Adrien E Desjardins

**Affiliations:** 1 William Harvey Cardiovascular Research Institute, Queen Mary University of London and Barts Heart Centre, London EC1A 7BE, UK; 2 Wellcome/EPSRC Centre for Interventional and Surgical Sciences, University College London, Charles Bell House, 67-73 Riding House Street, London W1W 7EJ, UK; 3 UCL Centre for Materials Research, Department of Chemistry, University College London, London WC1H 0AJ, UK; 4 Department of Electronic and Electrical Engineering, University College London, London WC1E 7JE, UK

**Keywords:** cardiac, medical devices, optoacoustic, photoacoustic, ultrasound

## Abstract

High-frequency ultrasound imaging can provide exquisite visualizations of tissue to guide minimally invasive procedures. Here, we demonstrate that an all-optical ultrasound transducer, through which light guided by optical fibers is used to generate and receive ultrasound, is suitable for real-time invasive medical imaging *in vivo*. Broad-bandwidth ultrasound generation was achieved through the photoacoustic excitation of a multiwalled carbon nanotube-polydimethylsiloxane composite coating on the distal end of a 300-μm multi-mode optical fiber by a pulsed laser. The interrogation of a high-finesse Fabry–Pérot cavity on a single-mode optical fiber by a wavelength-tunable continuous-wave laser was applied for ultrasound reception. This transducer was integrated within a custom inner transseptal needle (diameter 1.08 mm; length 78 cm) that included a metallic septum to acoustically isolate the two optical fibers. The use of this needle within the beating heart of a pig provided unprecedented real-time views (50 Hz scan rate) of cardiac tissue (depth: 2.5 cm; axial resolution: 64 μm) and revealed the critical anatomical structures required to safely perform a transseptal crossing: the right and left atrial walls, the right atrial appendage, and the limbus fossae ovalis. This new paradigm will allow ultrasound imaging to be integrated into a broad range of minimally invasive devices in different clinical contexts.

## Introduction

High-frequency ultrasound imaging can provide an exquisite visualization of tissue to guide minimally invasive procedures, but it remains severely underutilized in clinical practice. Higher levels of spatial resolution are achieved at the expense of imaging depth, so the integration of ultrasound transducers into medical devices is often required to visualize tissue microstructures from within the body. Since its inception, ultrasound imaging has been achieved using electronic transducers. All-optical ultrasound transducers, which perform ultrasonic generation using pulsed light and optical reception of ultrasonic reflections from tissues, could serve as viable alternatives^1, 2, 3, 4, 5, 6, 7^.

Here, we demonstrate that all-optical ultrasound transducers can provide real-time interventional imaging to guide minimally invasive procedures. To bring all-optical ultrasound imaging to a point of clinical utility, three advances were required: sufficient sensitivity to reflected ultrasound to provide soft-tissue contrast at centimeter-scale depths in the presence of tissue and device motion; the integration of sensing elements into devices in a manner that preserves current clinical workflows; and an *in vivo* demonstration through which clinically relevant information can be acquired. We are the first to report the successful attainment of these requirements.

As an illustration of the clinical utility of real-time *in vivo* all-optical ultrasound imaging, we adapted our technique to be used during cardiac transseptal puncturing. During this widely performed procedural step, a needle is inserted into the right atrium of the heart via the femoral vein, where it is used to puncture the foramen ovale to gain access to the left atrium for therapeutic intervention^8, 9^. In current practice, transseptal puncturing is principally guided by X-ray fluoroscopy, which does not provide soft-tissue contrast; cost sensitivities prevent the routine use of conventional intracardiac echocardiography (ICE) outside of the USA^10^.

**Figure 1:**
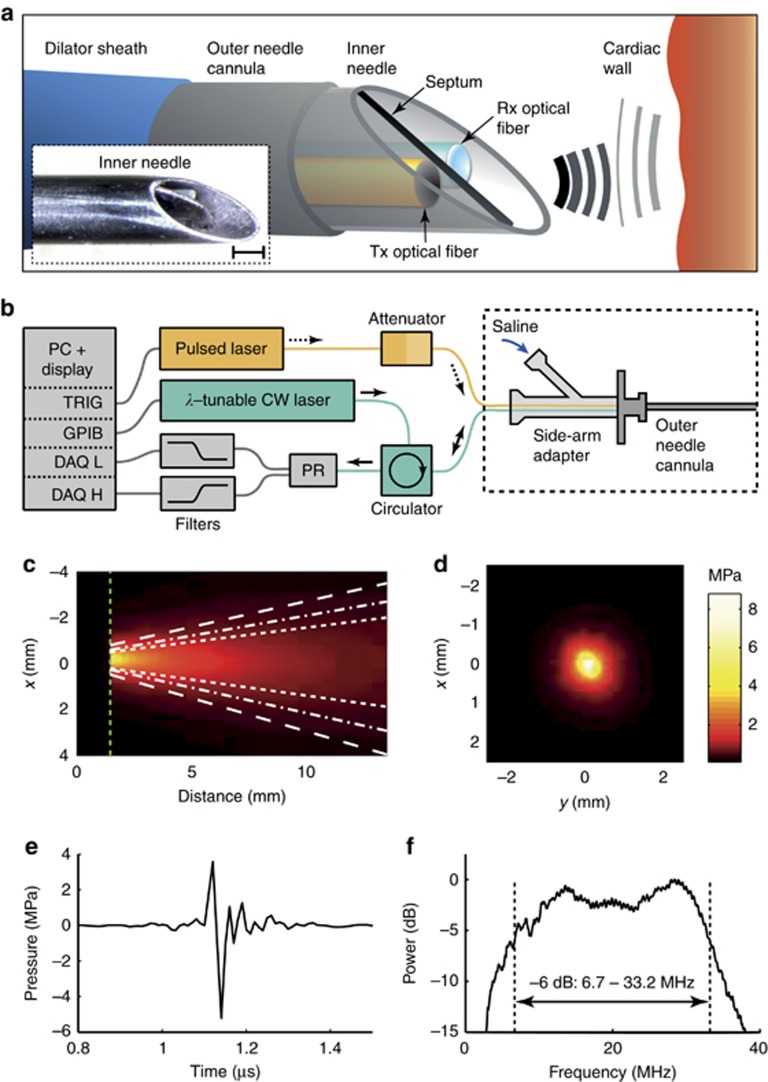
System for all-optical ultrasound imaging through a needle (**a**). The sharp inner needle (schematic and inset photo) used to puncture the cardiac septum to gain access to the left atrium can be safely recessed within a blunt outer needle cannula. After puncturing, the dilator sheath is advanced over the needle into the left atrium. The probe includes two optical fibers positioned within the inner needle for pulse-echo ultrasound imaging: one for transmission (Tx) with the delivery of pulsed excitation light to an optically absorbing coating and one for reception (Rx) with the delivery of continuous-wave (CW) light to a Fabry–Pérot cavity. Acoustic isolation between the Tx and Rx fibers is provided by a thin metal septum. Scale bar, 500 μm. The corresponding console (**b**) delivers pulsed excitation to the Tx fiber and CW light from a wavelength-tunable laser to the Rx fiber. Reflections from the Rx fiber are detected with a photoreceiver (PR) through an optical circulator. Low-frequency PR signals are used to determine the optimal wavelength tuning of the CW laser; high-frequency PR signals are processed to generate ultrasound images and are displayed in real-time. Spatially resolved transmitted ultrasound as measured in a plane parallel to the optical fiber axis (**c**) had a divergence angle (FWHM) of 23° (dashed-dotted white lines). The divergence was smaller (15.2°) for frequencies of 20–40 MHz (short dashed white lines) and larger (29.9°) for frequencies of 2.5–20 MHz (long dashed white lines). Spatially resolved transmitted ultrasound measured in a plane perpendicular to the optical fiber axis 1.5 mm from the fiber tip (green dashed lines in **c**) was circularly symmetric (**d**). Time-resolved transmitted ultrasound measured on-axis at 1.5 mm from the fiber tip was predominantly bipolar (**e**) with a broad bandwidth (−6 dB) of 26.5 MHz (**f**). DAQ, data acquisition; DAQ H, DAQ for high-frequency PR signal; DAQ L, DAQ for low-frequency PR signal; GPIB, general purpose interface bus; TRIG, trigger.

In the present study, all-optical pulse-echo ultrasound imaging was used to visualize cardiac tissue directly ahead of the distal end of a transseptal puncture needle. This was achieved using two optical fibers within a custom inner transseptal needle small enough to be fitted within a commercial outer needle cannula (Figure 1a). Ultrasound waves were generated photoacoustically through light pulses delivered to an optically absorbing composite coating on the distal end of one of the optical fibers. Ultrasound waves reflected from tissue were detected through a Fabry–Pérot cavity positioned on the distal end of the other optical fiber. Depth scans were concatenated across time and displayed in real-time as M-mode ultrasound images.

## Materials and methods

### Optical transmitter and receiver

The multi-mode optical fiber used for ultrasound generation had silica core/cladding diameters of 300/318 μm (CeramOptec GmbH, Germany). At the distal end, the polyimide coating was removed with hot sulfuric acid to expose the cladding, and the optical fiber was then cleaved perpendicular to its axis. Ultrasound was generated with a multiwalled carbon nanotube (MWCNT)-polydimethylsiloxane (PDMS) coating^11, 12^. The coating was applied by dip-coating an organogel that was prepared as follows: MWCNTs (6–9 nm × 5 μm, Sigma Aldrich, UK) were functionalized using an oleylamine-functionalized pyrene as described previously^13^ and were dispersed in xylene (14 mg ml^−1^). The xylene dispersed functionalized MWCNTs were sonicated with acetone (VWR, UK) using a 2:1 MWCNT-xylene:acetone ratio and were allowed to rest overnight, forming a gel^14^. After dip-coating with the gel, the fibers were left to stand for ~1 h to allow the evaporation of solvents prior to dip-coating with PDMS (Nusil MED1000, Polymer Systems Technology, UK) diluted in xylene (1 g PDMS: 1.8 ml xylene).

The optical fiber used for ultrasound reception was of single-mode (SMF-28) with core/cladding diameters of 8/125 μm. A Fabry–Pérot (FP) cavity was created by dip-coating with an optically transparent polymer, as previously described^15^. Two dielectric mirrors were deposited before and after dip-coating the polymer: one on the optical fiber end face and a second on the outer surface of the polymer. Both mirror reflectivities were nominally 98% in the range of 1500–1600 nm. The FP cavity was coated in a protective layer of parylene C of approximately 5 μm.

### Cardiac needle

Transmitting and receiving fibers were housed within the custom inner needle of a coaxial transseptal needle. This inner needle was fabricated from a stainless steel hypotube with nominal inner/outer diameters of 0.889/1.08 mm (19X; MicroGroup, USA) and a length of 78 cm. The distal end was beveled at 60° relative to the hypotube axis. When fully extended within a commercial 17 gauge Endry’s transseptal puncture outer needle cannula, its distal end protruded by 10 mm. A beveled septum that provided acoustic isolation between the two optical fibers used for transmission and reception was positioned within the inner needle at the distal end. With a length of 10 mm along the hypotube axis and a width equal to the inner diameter of the hypotube, the septum was laser cut from 0.1-mm-thick stainless steel. The septum did not extend beyond the bevel surface of the needle.

The optical fibers were affixed to the inner wall of the hypotube 30 mm from the distal end. To do so, a small opening was created in the hypotube. Once the fibers were positioned within the needle, a small bolus of sealing wax was deposited through the opening so that it held fibers adjacent to the inner wall of the hypotube. This left sufficient space within the hypotube to permit fluid injections through the inner needle; fluid was prevented from flowing out from the window through the outer needle cannula in close apposition to the inner needle (Figure 1a).

At the proximal end, a side-arm adapter (Cook Medical, UK) allowed for fluid to be injected through the inner needle via the side Luer lock port and for optical fibers to exit the needle through the other port. The inner needle had a Luer lock at its proximal end to connect it with the outer needle cannula. Outside of the needle, optical fibers from the inner needle were contained within Tefzel tubing with adhesive-lined heat shrink tubing at the ends for strain relief.

### Console

The main components of the console were lasers for interrogating the all-optical ultrasound probe, electronics for receiving and digitizing the ultrasound signal, and a workstation with custom software (Figure 1b). For ultrasound generation, pulsed excitation light with a wavelength of 1064 nm, a pulse width of 2 ns, and a pulse energy level of 20 μJ was delivered into the ultrasound generating optical fiber from an Nd:YAG laser (SPOT-10-500-1064, Elforlight, UK). This laser was externally triggered at 50 Hz. For ultrasound reception, continuous-wave light from a tunable laser (Tunics T100S-HP CL, Yenista Optics, France) operated at an output power level of 9 mW in the wavelength range of 1520–1570 nm was first attenuated by 10 dB with a fiber-optic coupler and then delivered to the ultrasound reception optical fiber through a circulator. The reflected signal was received by a photoreceiver with custom electronics that provided two output signals: low (<50 kHz) and high-frequency (>500 kHz) components of the photodetector signal. To prevent aliasing during digitization, a low pass analog filter with a cut-off of 48 MHz was applied to the high-frequency component. The low-frequency component was digitized at 16 bits with a sample rate of 1 MS s^−1^ (PCI-6251, National Instruments, UK) and was used to record the FP transfer function. The wavelength of the tunable laser was adjusted to correspond to a local maximum of the derivative of the FP transfer function to optimize sensitivity^16^. The high-frequency component was the ultrasound signal that originated from variations in the reflectivity of the FP cavity produced by impinging ultrasound waves; it was digitized at 14 bits at a sample rate of 100 MS s^−1^ (PCI-5142, National Instruments, UK).

### Signal and image processing

The custom software, written in LabVIEW (National Instruments, USA), controlled data acquisition, performed processing, and provided a real-time display of the ultrasound signal as an M-mode image at 50 Hz. Offline processing was performed using the same algorithms implemented in Matlab (Mathworks, USA). The software also continuously saved data to allow for offline processing. Noise removal involved bandpass filtering and ultrasonic cross-talk modeling. First, low- and high-pass frequency filters with cut-offs of 1.5 and 45 MHz (4th order Butterworth) were applied. Next, ultrasound signals were processed to remove ultrasonic cross-talk that arose from ultrasound propagation directly from the generation fiber to the reception fiber, which varied with time. This cross-talk removal algorithm has been described in other work^4^. In brief, each scan was fit with a general linear model of 3 components: a local average obtained from 20 scans, the derivative of the local average to allow for temporal offsets, and a constant term. The modeled cross-talk for each scan was subtracted from the signals.

After noise and cross-talk removal, digital time-gain compensation was applied. This was achieved by multiplying the signal by the following gain factor, *g*(*i*):


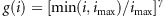


where *i* is the sample index of the signal (*i*=1, 2, …). Parameters *i*_max_ and *γ* were empirically designated as 700 and 2.5, respectively. The envelope of the signal was then obtained with the absolute value of the Hilbert transform. The enveloped signals were logarithmically transformed, concatenated across time, and displayed in real-time as an M-mode image. A two-dimensional median filter with a 3 × 3 window size was used to suppress speckle noise. Conversion from sample indices to the depth from the needle tip was performed using a sound speed of 1540 m s^−1^.

### *In vivo* imaging

Pigs were housed in accordance with UK Home Office guidelines relating to animal welfare, and our work was conducted within the scope of UK Home Office License PPL 70/7765 of the Northwick Park Institute for Medical Research (NPIMR, London, UK). The protocol was reviewed and approved by the NPIMR Animal Welfare and Ethical Review Body. Imaging was performed on two pigs (45 kg each). Pigs were placed under terminal anesthesia and maintained with isoflurane; they were continuously monitored. Transseptal puncture needles with all-optical ultrasound imaging were inserted through a 10 French (F) introducer sheath (Flexor, Cook Medical, USA) via the femoral vein. A second 10 F introducer sheath was used to introduce an intracardiac echocardiography (ICE) catheter (AcuNav 8 F, Siemens, USA). Transseptal puncture needles were introduced through a dilator sheath (Flexor, Cook Medical) to the superior vena cava over a 0.889 mm (0.035’) diameter guidewire.

## Results and discussion

The all-optical ultrasound transducer used in this study was the first to be developed and used for interventional imaging. Its integration into the inner transseptal puncture needle presented several challenges: mitigating acoustic reverberations within the device, adhering to space constraints imposed by the use of the commercial outer needle cannula, and allowing for fluid injections through the inner needle. Further, its use during an invasive procedure required video-rate image acquisition and real-time display. Here, optically generated ultrasound pulses generated peak-to-peak pressure levels of 8.8 MPa at 1.5 mm from the distal end of the fiber with a −6 dB bandwidth of 26.5 MHz and an angular divergence (full-width at half-maximum, FWHM) of 23° (Figure 1c–1f). This angular divergence increased with decreasing frequency: it was 29.9° over a frequency range of 2.5–20 MHz and 15.2° over a range of 20–40 MHz. By contrast, the fiber-optic ultrasound generator with a MWCNT-PDMS coating developed by Colchester *et al.*^13^ used for benchtop all-optical ultrasound imaging with synthetic aperture reconstruction generated a peak-to-peak pressure level of 1.96 MPa at 1.5 mm, a −6 dB bandwidth of 15 MHz, and an angular divergence (FWHM) of 29°. For interventional imaging with a single transducer, lower levels of divergence help increase imaging depths and improve lateral resolutions when synthetic aperture reconstruction is impractical due to tissue and device motion. The ultrasound generation performance compared favorably with that measured from the commercial electronic ICE probe (Supplementary Information; Supplementary Figs. S1 and S2; Supplementary Table S1).

**Figure 2:**
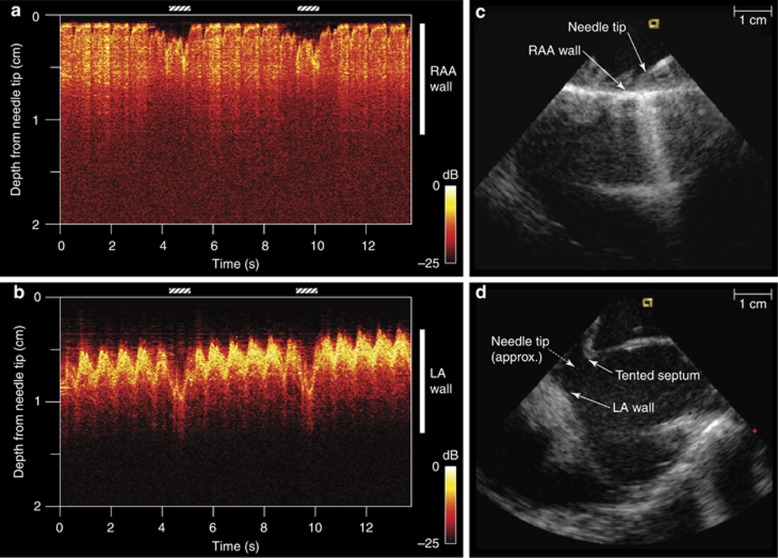
All-optical ultrasound imaging (M-mode) before (**a**) and after (**b**) the perforation of the cardiac septum. Corresponding needle tip positions were identified with a commercial intracardiac echocardiography catheter (**c** and **d**). With the needle tip positioned at the right atrial appendage wall, imaging depths extended more than 1 cm into tissue **a**. Cardiac motion, which manifested as slight deviations of the tissue surface relative to the needle tip, was more prominent during mechanical ventilation (diagonal bars). Immediately following perforation and entrance into the left atrium (LA), pronounced LA wall cardiac motion was readily apparent **b**.

**Figure 3:**
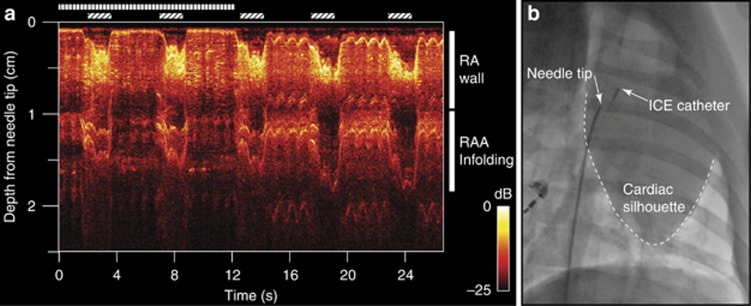
All-optical ultrasound imaging from the right atrium (RA) with depth scans shown longitudinally in time for M-mode imaging (**a**). The needle was pointed anteriorly and was initially held against the RA wall (vertical bars). Slight retraction was performed (>12 s), and pulsations of the RA wall became apparent (0.1–0.3 cm in depth from the needle tip). As mechanical ventilation was performed (diagonal bars), the resulting cardiac shifts produced changes in the apparent thickness of the RA wall. Right atrial appendage infolding and motion was visible beyond the RA wall. Conventional X-ray fluoroscopy imaging was performed concurrently (**b**).

Through-needle all-optical ultrasound images of multiple locations within a swine heart were obtained with needle tip locations verified via X-ray fluoroscopy and ICE. An ICE catheter was positioned within the superior right atrium, and agitated saline bubble contrast injection facilitated the identification of the needle tip. Cardiac tissue structures and their kinematics were visualized in real time (Figure 2). The cardiac wall could be distinguished at distances of greater than 2.5 cm from the needle tip, and the distance from the needle tip to the cardiac wall could readily be measured. Before transseptal puncturing, the right atrial appendage (RAA) wall was visualized at imaging depths of beyond 1 cm (Figure 2a). Subsequently, the far left atrial wall exhibited pronounced systolic motion (Figure 2b). This is of significant clinical relevance: inadvertent far-wall puncturing is a well-recognized risk, yet the true distance between a needle tip and atrial wall is difficult to determine through the indirect view provided when using other modalities (for example, catheter-based ICE). Variations in the positioning of the endocardial surface resulting from both systolic contractions and respiration were apparent, as were those of deeper structures and contralateral cardiac walls. The movements of complex infoldings of the RAA were visible with the through-needle ultrasound stylet pointed anteriorly (Figure 3); the advancement of the needle at this point would risk extracardiac puncturing. An illustration of the relationships of this device to these anatomical structures is provided in Supplementary Information and Supplementary Fig. S3.

**Figure 4:**
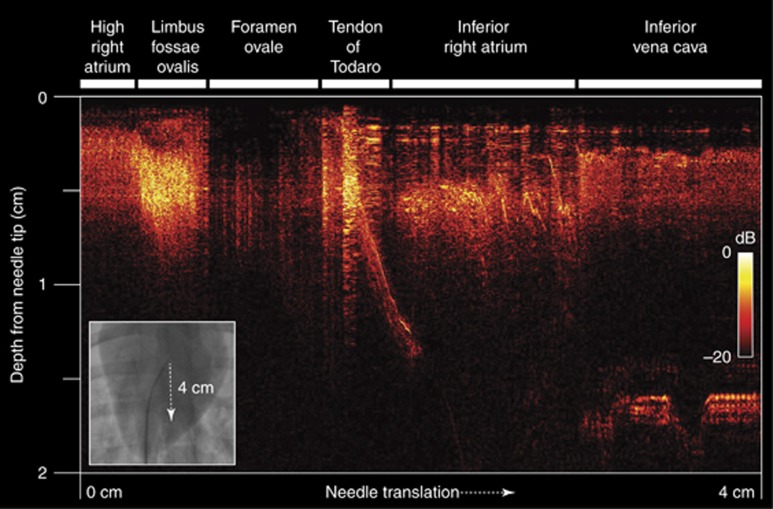
Two-dimensional all-optical ultrasound imaging (B-Mode) acquired during the manual translation of the needle tip across a distance of 4 cm. As the needle tip progressed from the high right atrium to the inferior vena cava, the thin foramen ovale manifested as a hypoechoic region between the thick limbus fossae ovalis and the tendon of Todaro (with a diagonal artifact from the ICE catheter and sheath). X-ray fluoroscopic imaging was acquired concurrently (inset).

In clinical practice, a needle ‘drag-back’ is often performed to identify a foramen ovale via X-ray fluoroscopy^8^. Through this maneuver, the needle is manually translated from superior to inferior parts of the right atrium (Supplementary Information and Supplementary Fig. S4). A slight deflection of the needle tip can be observed as the needle tip passes over the thick ridge of the limbus fossae ovalis, but this movement is often ambiguous. We performed such a maneuver via concurrent through-needle ultrasound imaging and extended translation into the inferior vena cava (Figure 4). Variations in the thicknesses of tissues in front of the tip were observed. An apparent tissue thickness of greater than 0.9 cm was observed at the limbus fossae ovalis; immediately beyond this point, the thickness decreased, and the ultrasound reflectivity decreased abruptly at the foramen ovale. The thickness then increased again, reaching 1.5 cm at the tendon of Todaro.

Several challenges remain to be addressed for the translation of all-optical ultrasound transducers into medical devices used in clinical practice. First, it must be ensured that heat generated on a coating does not elevate the temperature of surrounding blood to harmful levels. The all-optical ultrasound transducer described here has an efficiency of ~0.5%, as estimated through spatially resolved pressure measurements (Figure 1c) and from the spatial integration of energy computed at each location via Biagi *et al.*’s (Equation (8)) method^17^. The remaining energy level of an excitation light pulse, which is not converted into ultrasound waves, transiently elevated the temperature of the coating. On the basis of the assumption that the thermal behavior of this coating is dominated by the pure PDMS region (specific heat: 1.46 J g^−1^ K^−1^; estimated coating thickness: 20 μm), the temperature elevation reached approximately 10 °C. In future versions of the transducer, the peak temperature experienced by adjacent tissues could be significantly lowered by means of a polymer coating. With the pulse energy and repetition rate used here, the time-averaged heating rate is only 0.995 mW, and blood flow will reduce heat in the vicinity of the needle tip. An additional translation challenge involves the biocompatibility of the MWCNT-PDMS composite, which was not tested in this study. The integration of MWCNTs within PDMS should prevent their contact with biological tissues. A thin barrier coating with a biologically inert polymer such as parylene, as applied to the outer surface of the FP cavity, would likely ensure biocompatibility.

Given their small lateral dimensions, flexibility, and immunity to electromagnetic interference, fiber optics tend to be very well suited to sensing from within medical devices, and this is particularly the case for those used in cardiovascular medicine^18^. In future devices, fiber optics in the all-optical ultrasound transducer presented here could be used to deliver light to tissues for concurrent photoacoustic imaging^19, 20, 21, 22, 23, 24^ or for optical coherence tomography^25, 26, 27^. The omni-directionality of the ultrasound receiver^15^ is well suited to receiving pulses from distant ultrasound imaging probes to track the positioning of the needle tip^28^.

All-optical ultrasound transducers may serve as valuable alternatives to electronic transducers in many cases, with levels of optimality generally depending on several factors such as depths, sizes, and angulations of the relevant structures to be imaged. With both transducer types, improvements in sensitivity yield increases in imaging depth; these increases can also be achieved at the expense of decreased spatial resolutions. Electronic transducer sensitivity levels tend to be improved by increasing the element size, which also reduces beam divergence. However, space available for transducers can be extremely limited within invasive medical devices. These constraints, along with the practicalities and complexities associated with incorporating electromagnetic shielding and integrated circuits, significantly limit the integration of electronic transducers into invasive medical devices.

In terms of imaging depths and spatial resolutions, the all-optical ultrasound transducer used in this study performed favorably with respect to electronic counterparts with broadly comparable frequency ranges that provided forward visualizations from the distal ends of needles and catheters^29, 30, 31, 32, 33^. For instance, Chiang *et al.*^30^ integrated an electronic transducer with a diameter of 0.5 mm and a center frequency of 40 MHz (−6 dB fractional bandwidth of 50%) into an epidural needle and reported an imaging depth of 10 mm in soft-tissue *in vivo* and an axial resolution of 150 μm. Similarly, in Wright *et al.*^29^, forward-viewing catheters (3.3 mm outer diameter; 10 F) with single transducers that were operated in the frequency range of 25–33 MHz allowed for the intracardiac visualization of myocardial tissues to depths of 5–10 mm *in vivo*. With all-optical ultrasound transducers, optimizations for biological tissues could readily be performed in ways that are not possible with electronic transducers. As optical ultrasound transmitters are non-resonant, the central frequency of transmitted ultrasound can be varied by changing the pulse duration of excitation light. The same transmitter can thus provide both low-frequency, high-depth imaging and high-frequency, low-depth imaging^6^. Likewise, the responsiveness, sensitivity and directionality of a Fabry–Pérot ultrasound receiver can be altered by changing the shape and thickness of the cavity^15^.

## Conclusions

In summary, we present a novel platform for performing pulse-echo ultrasound imaging using fiber optics integrated within a clinical needle through the photoacoustic excitation of a nanocomposite coating for generation and a high-finesse Fabry–Pérot cavity for reception. Our study is, to the best of our knowledge, the first to prove the viability of all-optical ultrasound methods for *in vivo* medical imaging and for dynamic imaging from within a beating heart for procedural guidance. The all-optical ultrasound transducers used in this study are ideally suited to interventional imaging, as they can deliver high resolutions at significant depths from within single-use medical devices. In the context of transseptal punctures, they yield a degree of soft-tissue contrast that is unattainable through X-ray imaging to visualize the correct location for crossing the septum and incorrect locations for avoiding complications. The advances presented here can readily be extended to a broad range of clinical and preclinical devices to generate ultrasound images of areas of the body for which they were previously unavailable.

## Author contributions

MCF, IPP, and AED conceived of and performed the study and wrote the paper. Transmitter and receiver coatings were conceived of and constructed by SN, RJC, EZZ, PCB, IPP and AED. The inner needle assembly was created by CAM. The software was written by RJC and AED. Advice on study protocols and assistance with the experiments was provided by RJS and SO. All of the authors discussed the results and commented on the manuscript.
