# Supplementary material for: Through-needle all-optical ultrasound imaging in vivo: a preclinical swine study
Source: Light Sci Appl. 2017 Dec 1;6(12):e17103–. doi: 10.1038/lsa.2017.103 (PMC6062020; doi:10.1038/lsa.2017.103)
Supplement: Supplementary Information [file lsa2017103x1.docx]

**Supplementary Information for**

**Through-Needle All-Optical Ultrasound Imaging *In Vivo*: A Pre-clinical Swine Study**

Malcolm C. Finlay^1^, Charles A. Mosse^2^, Richard J. Colchester^2^, Sacha Noimark^3^, Edward Z. Zhang^2^, Sebastien Ourselin^2^, Paul C. Beard^2^, Richard J. Schilling^1^, Ivan P. Parkin^3^, Ioannis Papakonstantinou^4^, Adrien E. Desjardins^2^

^1^William Harvey Cardiovascular Research Institute, Queen Mary University of London & Barts Heart Centre, London, United Kingdom

^2^Department of Medical Physics and Biomedical Engineering, University College London, London, United Kingdom

^3^UCL Centre for Materials Research, Department of Chemistry, University College London, London, United Kingdom

^4^Department of Electronic and Electrical Engineering, University College London, London, United Kingdom

**Ultrasound characterisation: Transseptal puncture (TSP) needle**

Optically-generated ultrasound transmissions by the transseptal puncture (TSP) needle were measured with a 75 µm calibrated needle hydrophone (Precision Acoustics, UK). They were measured in two planes: one orthogonal to the optical fibre axis; the other, parallel to and intersecting with the optical fibre axis. Measurements in the orthogonal plane were acquired across a square grid centred on the fibre axis at a distance of 1.5 mm from the distal end of the optical fibre, with 200 × 200 points and a step size of 50 µm. Measurements in the parallel plane were acquired across a rectangular grid, with 100 × 200 points and a step size of 100 µm. The short sides of the grid were centred on the fibre axis, with a separation of 1.5 mm between the grid and the distal end of the fibre. The hydrophone signals were digitised at 14 bits with a sample rate of 100 MS/s (PCI-5142, National Instruments, UK). Measurements from the orthogonal and perpendicular planes are provided in Figure 1C and Figure 1D, respectively.

**Ultrasound characterisation: ICE catheter**

Electronically-generated ultrasound transmissions by the intracardiac echocardiography (ICE) catheter (AcuNav 8F, Siemens, USA) and the corresponding console (Cypress, Siemens, USA) were measured in-plane to obtain peak-to-peak pressures and bandwidths, and out-of-plane to calculate the beam divergence.

In-plane measurements were performed with a 200 µm diameter calibrated needle hydrophone (Precision Acoustics, UK). During these measurements, the ICE console performed continuous M-mode imaging in either the High or Low frequency mode. The hydrophone was centred within the ICE imaging plane, at three distances of 5, 10, and 20 mm from the catheter (as measured by direct observation of the hydrophone with ICE imaging). The hydrophone signals were triggered by the rising slope of the received ultrasound signals. The ultrasound signals were digitised at 14 bits with a sample rate of 250 MS/s (M4i.4420-x8, Spectrum, Germany). The acquisition time windows were 16 ms in duration. An ultrasound pressure waveform and the corresponding bandwidth for a hydrophone-catheter distance of 5 mm are provided in Figure S1a and Figure S1b, respectively. No discernible differences between the ultrasound bandwidths during M-mode and B-mode imaging were observed (data not shown).

Out-of-plane measurements were performed with a custom built, omnidirectional concave Fabry-Pérot hydrophone ^1^. During these measurements, the ICE console was set to perform M-mode imaging in the High frequency mode. Measurements were acquired within a plane that was perpendicular to the catheter axis and approximately centred with respect to the linear array, across a square grid with 151 × 151 points and a step size of 200 µm that was 0.5 mm from the catheter at its closest point. The hydrophone was interrogated using the same system used to receive ultrasound at the distal end of the TSP needle *in vivo* (*cf*. Methods section) and the acquisition was triggered off the rising slope of the received ultrasound. The in-plane measurements acquired with the 200 µm diameter needle hydrophone were used to calibrate the out-of-plane measurements, using the region of overlap in which the grid intersected the ICE imaging plane. The resulting ultrasound beam pattern is a maximum amplitude projection of the ultrasound signals recorded at each location [Figure S1c].

The characteristics of the ultrasound transmitted optically by the TSP needle and electronically by the ICE catheter are compared in Table S1.

**Figure S1 Electronically-generated ultrasound by the intracardiac echocardiography (ICE) catheter.** **(a)** Time-resolved transmitted ultrasound pulse, as measured in-plane at 5 mm from the ICE catheter (AcuNav, Siemens, USA) during M-mode imaging. **(b)** Power spectrum corresponding to the data in (a), with a -6 dB bandwidth of 5.5 MHz (dashed lines). **(c)** Spatially resolved peak pressures of the transmitted ultrasound, as measured in a plane perpendicular to the ICE imaging plane, during M-mode imaging with the ICE catheter. Measurements were performed at distances greater than 5 mm from the ICE catheter (dashed line). The location at which the data in (b) and (c) were acquired is indicated with a green cross.


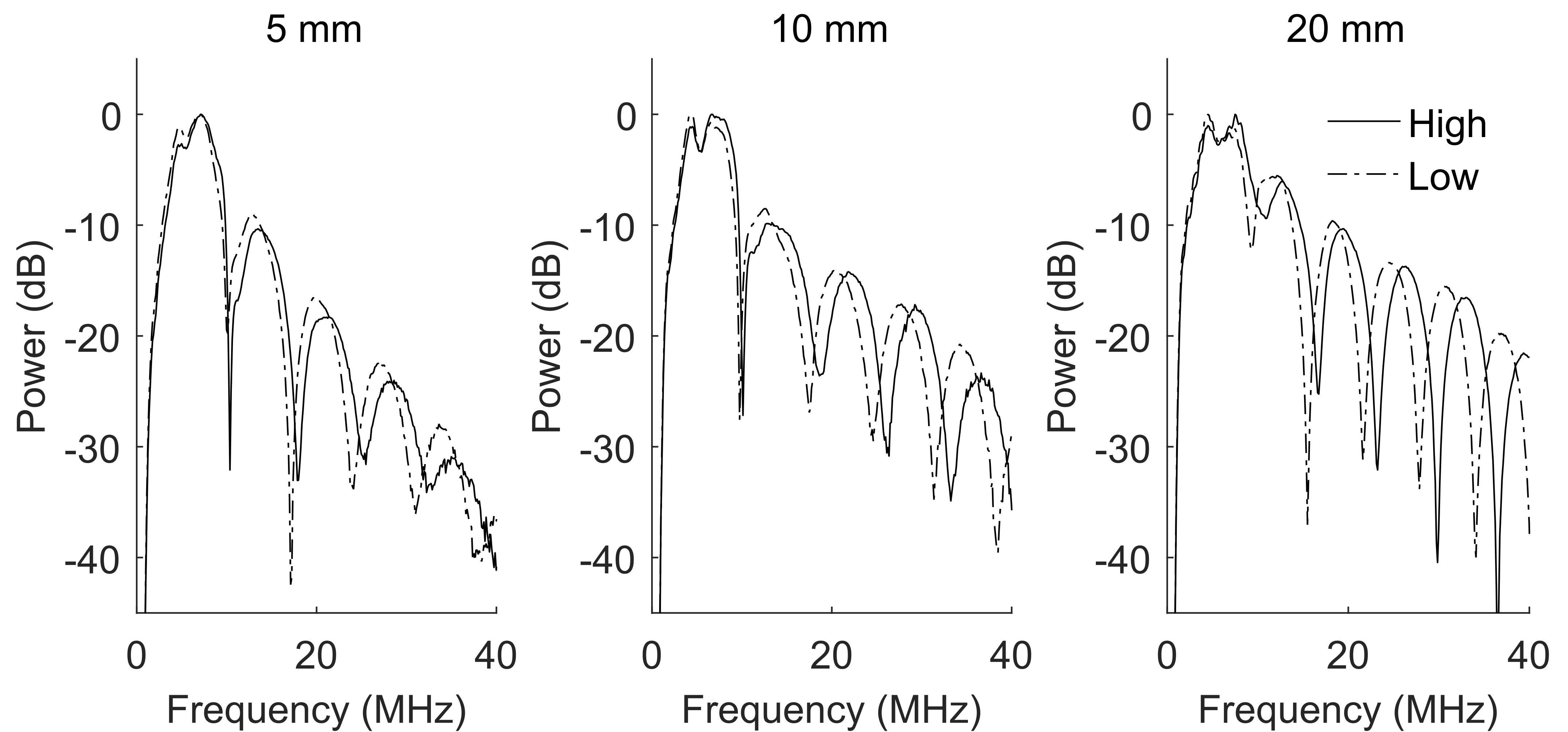


**Figure S2 Power spectra of the ultrasound that was electronically-generated by the intracardiac echocardiography (ICE) catheter.** These measurements were performed during M-mode imaging with the catheter (AcuNav, Siemens, USA), as measured in-plane at distances of 5 mm (left), 10 mm (centre), and 20 mm (right). For each, measurements were acquired with the High and Low frequency imaging modes of the console (Cypress, Siemens, USA).

|  | **Electronic Ultrasound ICE catheter** | **All-Optical Ultrasound  TSP needle** |
| --- | --- | --- |
| **Device diameter** | 2.7 mm (8 French) | 1.08 mm |
| **Number of elements** | 64 (nominal) | 1 |
| **Aperture** | 7 mm (nominal) | 0.58 mm |
| **Centre frequency** | 7 MHz | 20 MHz |
| **Bandwidth (-6 dB)** | 5.5 MHz | 26.5 MHz |
| **Peak-to-peak pressure  at 5 mm from probe** | 5.6 MPa | 1.6 MPa |
| **Beam divergence** | 19° (out-of-plane) | 23° |
| **Beam width (FWHM) at 5 mm from probe** | 12 mm (out-of-plane) | 2.4 mm |

**Table S1 Comparison of the intracardiac echocardiography (ICE) catheter and the transseptal puncture (TSP) needle.**

**
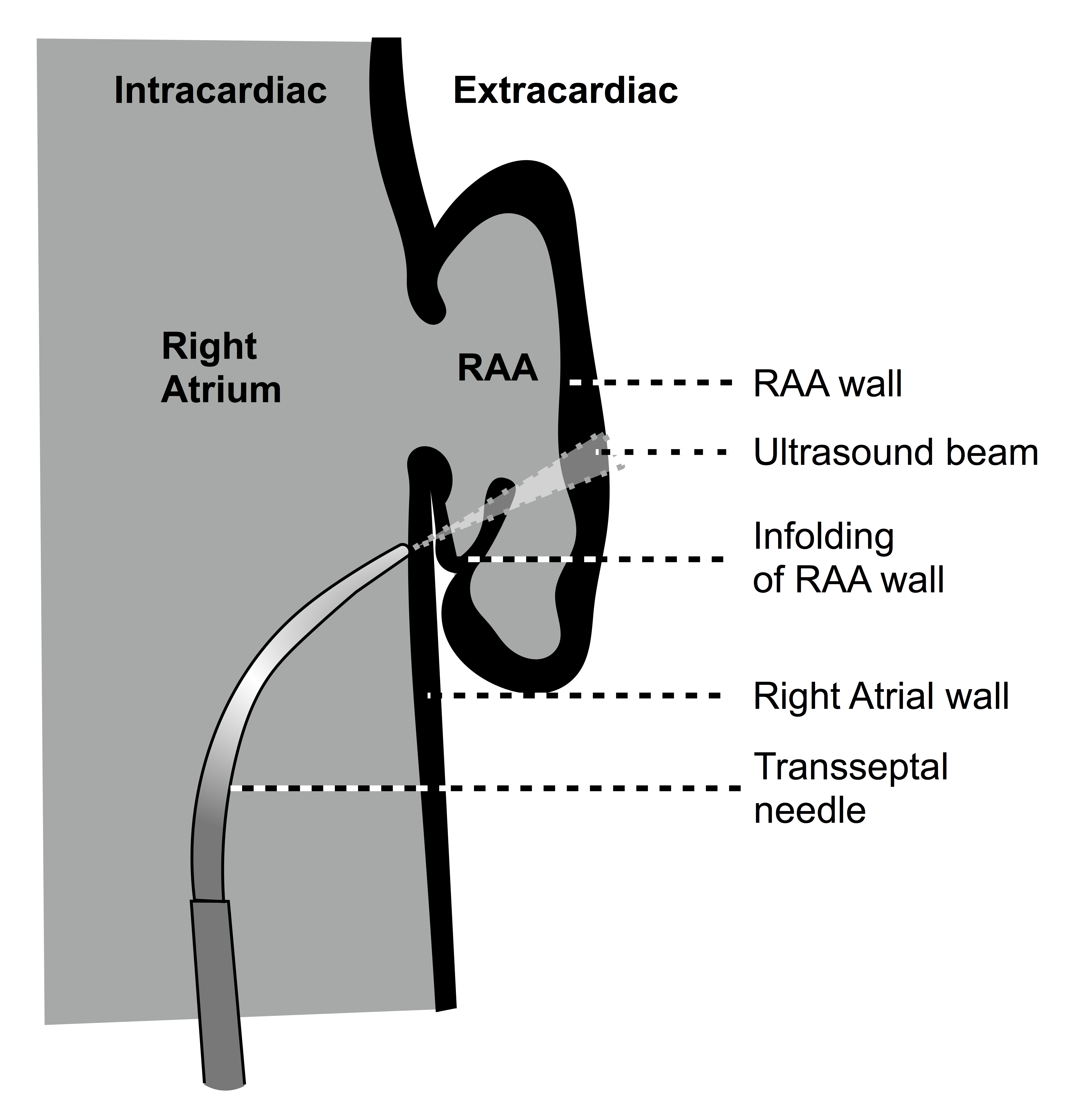
**

**Figure S3. Figurative representation of the estimated position of the transeptal needle tip within the beating swine heart during acquisition of the M-mode all-optical ultrasound image shown in Figure 3a.** This position was estimated with a combination of X-ray fluoroscopy [Figure 3b] and intracardiac echocardiography (ICE). In this interpretation, the transmitted ultrasound beam intersects both the right atrial (RA) wall and the right atrial appendage (RAA) infolding.

**
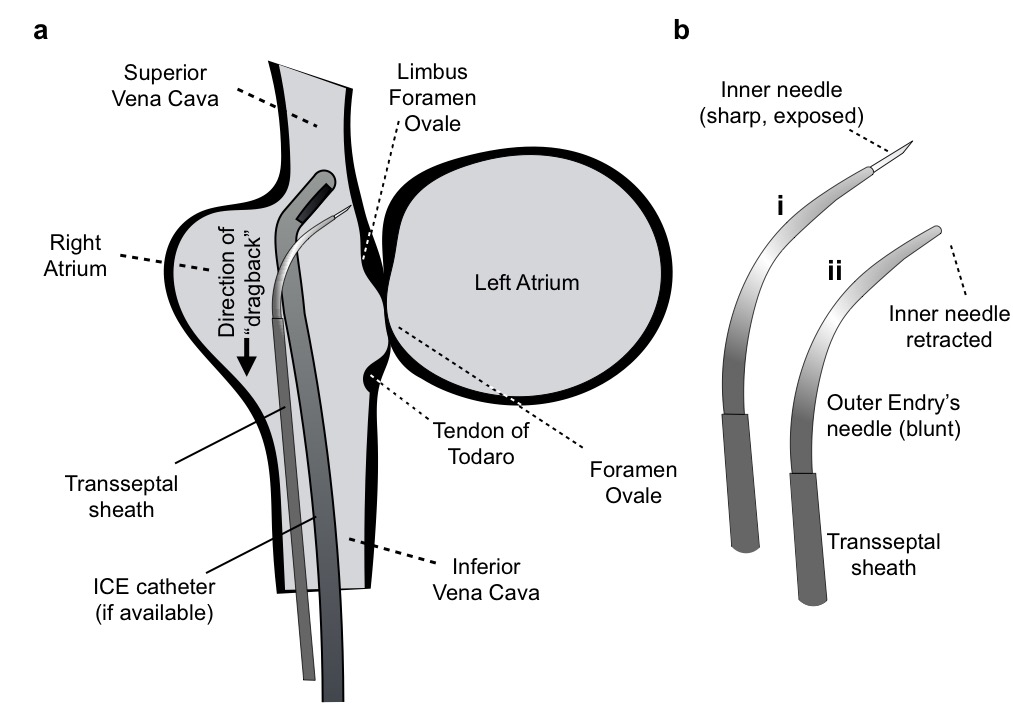
**

**Figure S4 Transseptal puncture. (a)** Figurative representation of salient anatomical features encountered during a “drag-back” manoeuvre of a transseptal puncture. In this representation, the anatomical features are oriented to coincide with the view obtained from a left anterior oblique projection on X-ray fluoroscopy. The drag-back is performed to position the tip of the transseptal needle adjacent to the foramen ovale, which is the location for puncture. During the drag-back, the transseptal sheath and the needles contained within it are pulled inferiorly (from up to down on this representation), with the needle tip tracing the path of the septal right atrial anatomy. In this study, an intracardiac echocardiography (ICE) catheter was also inserted into the right atrium from the IVC alongside the transseptal sheath. **(b)** During the drag-back, prior to puncture, the sharp inner needle is retracted relative to the blunt outer Endry’s needle (b, ii). When the foramen ovale is reached, the inner needle is exposed (b, i) for puncture. After puncture of the foramen ovale, in which the inner and outer needles pass from the right to the left atria, the transseptal sheath is advanced over the outer needle and the inner and outer needles are withdrawn; subsequently, the sheath serves as a conduit for other medical devices to access the left atrium. SVC: supervior vena cava; IVC: inferior vena cava.

**Reference**

1 Zhang EZ, Beard PC. Characteristics of optimized fibre-optic ultrasound receivers for minimally invasive photoacoustic detection. Proc SPIE 2015; **9323**: 932311
